# Application of fine-needle aspiration biopsy combined with multi-gene testing in the differential diagnosis of benign and malignant C-TIRADS 4 thyroid nodules

**DOI:** 10.3389/fmed.2026.1940501

**Published:** 2026-09-23

**Authors:** Yingying Yuan, Jinhua Zhao, Yi Ren, Li Sun, Jiejie Chen

**Affiliations:** 1 Department of Ultrasound, The Affiliated Huai’an No.1 People’s Hospital of Nanjing Medical University, Huai’an, Jiangsu, China; 2 Department of Nuclear Medicine, The Affiliated Shanghai First People’s Hospital of Nanjing Medical University, Shanghai, China; 3 Department of Thyroid and Breast Surgery, The Affiliated Huai’an No.1 People’s Hospital of Nanjing Medical University, Huai’an, Jiangsu, China

**Keywords:** BRAFV600E, C-TIRADS 4 category, diagnosis, fine-needle aspiration biopsy, multi-gene testing, Ras, RET/PTC, TERT

## Abstract

**Aim:**

To explore the clinical value of fine-needle aspiration biopsy (FNAB) combined with multi-gene testing (including BRAF^V600E^, TERT, RAS, and RET/PTC) in the differential diagnosis of benign and malignant thyroid Chinese Thyroid Imaging Reporting and Data System (C-TIRADS) 4 nodules and to compare its diagnostic performance with FNAB combined with single-gene testing.

**Methods:**

A total of 650 patients (650 nodules) with thyroid nodules who visited our hospital between January 2021 and December 2024 were included in this study. All patients underwent ultrasound-guided FNAB and multi-gene testing (including BRAF^V600E^, TERT, RAS, and RET/PTC). Using postoperative pathological findings as the “gold standard,” the diagnostic performance of ultrasound-guided FNAB and multi-gene testing for C-TIRADS 4 thyroid nodules was analyzed. The diagnostic efficacy of FNAB alone, each single-gene test alone, multi-gene testing alone, FNAB combined with each single-gene test, and FNAB combined with multi-gene testing in differentiating benign from malignant C-TIRADS 4 thyroid nodules was evaluated using receiver operating characteristic curves.

**Results:**

Among the 650 thyroid nodules, 432 were malignant, and 218 were benign according to postoperative pathology reports. FNAB combined with multi-gene testing demonstrated improved sensitivity compared with FNAB alone, with a sensitivity of 91.0%, specificity of 76.15%, and accuracy of 86.0%. The AUC of the combined method was 0.836, which was significantly higher than that of FNAB alone (0.781) and multi-gene testing alone (0.806) (*P* < 0.05). In FNAB-indeterminate nodules, FNAB combined with multi-gene testing achieved a sensitivity of 88.24% and specificity of 89.19%. In the sensitivity analysis reclassifying Bethesda IV as FNAB-positive, FNAB alone achieved a sensitivity of 83.6%, specificity of 62.8%, and accuracy of 76.6%, confirming that the combined testing strategy provided incremental diagnostic value.

**Conclusion:**

FNAB combined with multi-gene testing significantly improves the **sensitivity** for differentiating benign from malignant thyroid C-TIRADS 4 nodules. Compared with FNAB combined with single-gene BRAF^V600E^ testing, the multi-gene testing strategy offers improved sensitivity, particularly for detecting BRAF^V600E^-negative malignancies and FNAB-indeterminate nodules.

## Introduction

Thyroid nodules are among the most common endocrine disorders, and the incidence of thyroid malignancies has increased substantially worldwide (1, 2). Early and accurate differentiation between benign and malignant nodules is essential for appropriate treatment and prognosis (3, 4). The Chinese Thyroid Imaging Reporting and Data System (C-TIRADS) has standardized ultrasound-based risk stratification; however, C-TIRADS category 4 nodules remain diagnostically challenging because their malignancy risk varies widely, and ultrasound features frequently overlap between benign and malignant lesions (5, 6). Therefore, further clarification of the C-TIRADS 4 nodule status is a major clinical need.

Fine-needle aspiration biopsy (FNAB) is currently the standard minimally invasive method for the preoperative diagnosis of thyroid nodules (7). However, approximately 20% of nodules yield indeterminate cytology, such as Bethesda III/IV, limiting definitive diagnosis (8). Molecular testing, particularly BRAF^V600E^ mutation detection, improves diagnostic sensitivity; however, single-gene testing fails to detect malignancies lacking BRAF^V600E^, including follicular carcinoma and subsets of papillary carcinoma (9). Multi-gene panels, including BRAF^V600E^, TERT, RAS, and RET/PTC, may overcome these limitations and provide additional molecular information (10, 11).

Although multi-gene testing has shown promise, direct comparison between FNAB combined with single-gene and multi-gene testing in C-TIRADS 4 nodules remains limited. In particular, the clinical scenarios in which multi-gene testing adds the greatest incremental value, such as FNAB-indeterminate nodules and BRAF-negative malignancies, are not fully defined. Therefore, the present study aimed to evaluate the diagnostic performance of FNAB combined with multi-gene testing (BRAF^V600E^, TERT, RAS, and RET/PTC) for differentiating benign from malignant C-TIRADS 4 thyroid nodules and to compare this strategy with FNAB alone, multi-gene testing alone, and FNAB combined with each single-gene test. This study was not intended to promote routine molecular testing for all C-TIRADS 4 nodules; rather, it sought to clarify the incremental value of a multi-gene approach in selected challenging cases.

## Materials and methods

### Study population

A total of 650 patients with thyroid nodules (650 nodules) who visited our hospital between January 2021and December 2024 were included in this study. Among the participants, there were 162 men and 488 women. Their age ranged from 22 to 75 years, with a mean age of 48.32 ± 4.56 years. The nodule diameters ranged from 0.5 to 3.2 cm, with a mean diameter of 1.58 ± 0.32 cm. This study was approved by the Medical Ethics Committee of the Affiliated Shanghai First People's Hospital of Nanjing Medical University (Approval No. KY-2026-213-01). This study was conducted in accordance with the principles of the Declaration of Helsinki. Written informed consent was obtained from all participants before enrollment.

The inclusion criteria were as follows: (1) All thyroid nodules were clearly identified through pathological tissue examination. (2) All were classified as C-TIRADS 4 category thyroid nodules. (3) The nodules were visible on ultrasound examination, displaying characteristics such as irregular borders, hypoechoic appearance, and microcalcification.

The exclusion criteria were as follows: (1) Complicated with other severe thyroid diseases, such as hypothyroidism or hyperthyroidism. (2) Abnormal coagulation function. (3) Malignant tumors or metastases in other systems. (4) Pregnant women. (5) Mental disorders or inability to communicate normally.

### Methods

#### Ultrasound

The PHILIPS iU22 color Doppler ultrasound system (probe frequency ranging from 6.0 to 10.0 MHz) was selected. First, the size, boundary, shape, internal blood flow signal, internal echo, and calcification type of the thyroid nodules were observed. Blood flow and distribution within and around the lesion were observed.

#### FNAB

The patient was placed in a supine position with the neck fully exposed. An ultrasound interventional doctor conducted a routine ultrasound examination of the lesion site and confirmed the puncture path. Then, a routine iodophor disinfection was performed, a sterile towel was placed, and the movement of the needle tip was observed using real-time ultrasound. The needle core was withdrawn to create negative pressure, and the puncture needle was repeatedly withdrawn several times in different needle paths before being removed. A fine needle was inserted into a glass slide filled with air and immediately stained for microscopic examination. FNAB was performed by an ultrasound doctor with over 5 years of experience in needle puncture. All submitted sections were diagnosed by a pathologist with over 5 years of experience in thyroid cell pathology. According to the Bethesda standard (12), cytological diagnosis results are classified into six grades: Grade I, the specimen cannot be diagnosed or is unsatisfactory; Grade II, benign; Grade III, atypical cell of undetermined significance/follicular lesion of undetermined significance (AUS/FLUS); Grade IV, follicular neoplasm (FN) or suspected FN; Grade V, suspicious malignant tumor (SUSP); and Grade VI, malignant tumors. In the primary analysis, FNAB was considered positive for malignancy only when the cytology was classified as Bethesda grade V or VI. Bethesda IV nodules, which include follicular neoplasms that may ultimately prove malignant, were classified as FNAB-negative in the primary analysis. A sensitivity analysis reclassifying Bethesda IV as FNAB-positive was also performed.

#### Multi-gene testing

After the cytological smear was completed, the puncture needle connected to a 5 mL syringe was placed in an EP tube containing the multi-gene testing kit (including BRAF^V600E^, TERT, RAS, and RET/PTC genes) (Amoy Diagnostics Co., Ltd., Xiamen, China). The puncture needle was rinsed repeatedly. Post-puncture rinse fluid was collected from each patient, and DNA from the punctured nodule was extracted using a DNA extraction kit. The Ct value of the mutation or internal control signal was obtained by performing fluorescence quantitative PCR experiments. Based on the Ct value, the occurrence of a mutation d was determined. For each gene, a mutation signal Ct value of <28 was considered positive, and ≥28 was considered negative. Specifically, BRAF^V600E^ mutation detection targeted the c.1799T>A (p.V600E) hotspot; TERT promoter mutation detection targeted the C228T and C250T hotspots; RAS mutation detection targeted codons 12, 13, and 61 of NRAS, HRAS, and KRAS, respectively; and RET/PTC rearrangement detection targeted the most common fusion types, RET/PTC1 and RET/PTC3. The cytological results of FNAB were classified as Bethesda grades V and VI, as the diagnostic criteria for thyroid cancer, and positivity for any of the detected gene mutations was used as a diagnostic criterion for thyroid cancer.

#### FNAB combined with single-gene testing

For comparison, we evaluated the diagnostic performance of FNAB combined with each gene test. The diagnostic criteria for thyroid cancer were Bethesda grades V and VI or positivity for the specific gene being tested (BRAF^V600E^, TERT, RAS, or RET/PTC).

#### FNAB combined with multi-gene testing

The diagnostic criteria for thyroid cancer were Bethesda grades V or VI or positivity for any detected gene mutation (BRAF^V600E^, TERT, RAS, or RET/PTC). This logical OR (either FNAB or gene testing being positive) rule was consistently applied to all combined analyses. Under this rule, any nodule classified as FNAB-positive (Bethesda V/VI) was automatically classified as combined-test positive, regardless of the gene mutation status. Consequently, the false-positive count for the combined test could not be lower than that for FNAB alone.

### Outcome measures

Using postoperative pathological histological examination as the gold standard, this study analyzed the sensitivity, specificity, accuracy, positive predictive value, and negative predictive value of various diagnostic methods in distinguishing between benign and malignant thyroid C-TIRADS 4 nodules.

### Statistical analysis

Data were processed using SPSS version 26.0. Categorical data are presented as the number of cases (%), and differences between groups were compared using the chi-square test or Fisher's exact test. The Kappa test was used to analyze the consistency between each examination method and the pathological diagnosis. A receiver operating characteristic (ROC) curve was used to evaluate the diagnostic efficacy of the methods. Because all diagnostic indices were binary (positive/negative), each ROC curve represented a single operating point, and the AUC was used primarily as a summary measure of overall discrimination. The AUC values should be interpreted in conjunction with sensitivity and specificity, rather than as threshold-independent measures of accuracy. The diagnostic performances of FNAB alone, each single-gene test alone, multi-gene testing alone, FNAB combined with each single-gene test, and FNAB combined with multi-gene testing were compared. The DeLong test, which is appropriate for correlated ROC curves derived from the same cohort, was used to compare the AUC values of the different diagnostic methods. A pre-specified sensitivity analysis was performed by reclassifying Bethesda IV nodules as FNAB-positive to evaluate the robustness of the primary findings.

## Results

### Postoperative pathological results

All 650 thyroid nodules were surgically resected. Postoperative pathological results showed 432 malignant nodules (66.46%), including 386 papillary thyroid carcinomas (89.35%), 28 follicular thyroid carcinomas (6.48%), 12 medullary thyroid carcinomas (2.78%), and 6 anaplastic carcinomas (1.39%); and 218 benign nodules (33.54%), including 112 nodular goiters (51.38%), 68 thyroid adenomas (31.19%), 22 Hashimoto's thyroiditis (10.09%), and 16 subacute thyroiditis (7.34%).

### Baseline patient characteristics

Table 1 shows the baseline clinicopathological characteristics of the patients in this study. There were no statistically significant differences between the two groups in terms of age, sex, nodule diameter, and nodule location (*P* > 0.05), indicating comparability between the groups. The patient enrollment flowchart is shown in Figure 1.

**Table 1:** Baseline clinicopathological characteristics of patients.

Characteristic	Benign group (*n* = 218)	Malignant group (*n* = 432)	*t*/*χ*^2^-value	*P*-value
Age (years)	47.86 ± 4.32	48.55 ± 4.68	1.825	0.068
Sex			0.352	0.553
Male	52 (23.85)	110 (25.46)		
Female	166 (76.15)	322 (74.54)		
Nodule diameter (cm)	1.52 ± 0.28	1.61 ± 0.34	3.456	0.059
Nodule location			0.215	0.898
Left	98 (44.95)	192 (44.44)		
Right	106 (48.62)	214 (49.54)		
Isthmus	14 (6.43)	26 (6.02)		

**Figure 1:**
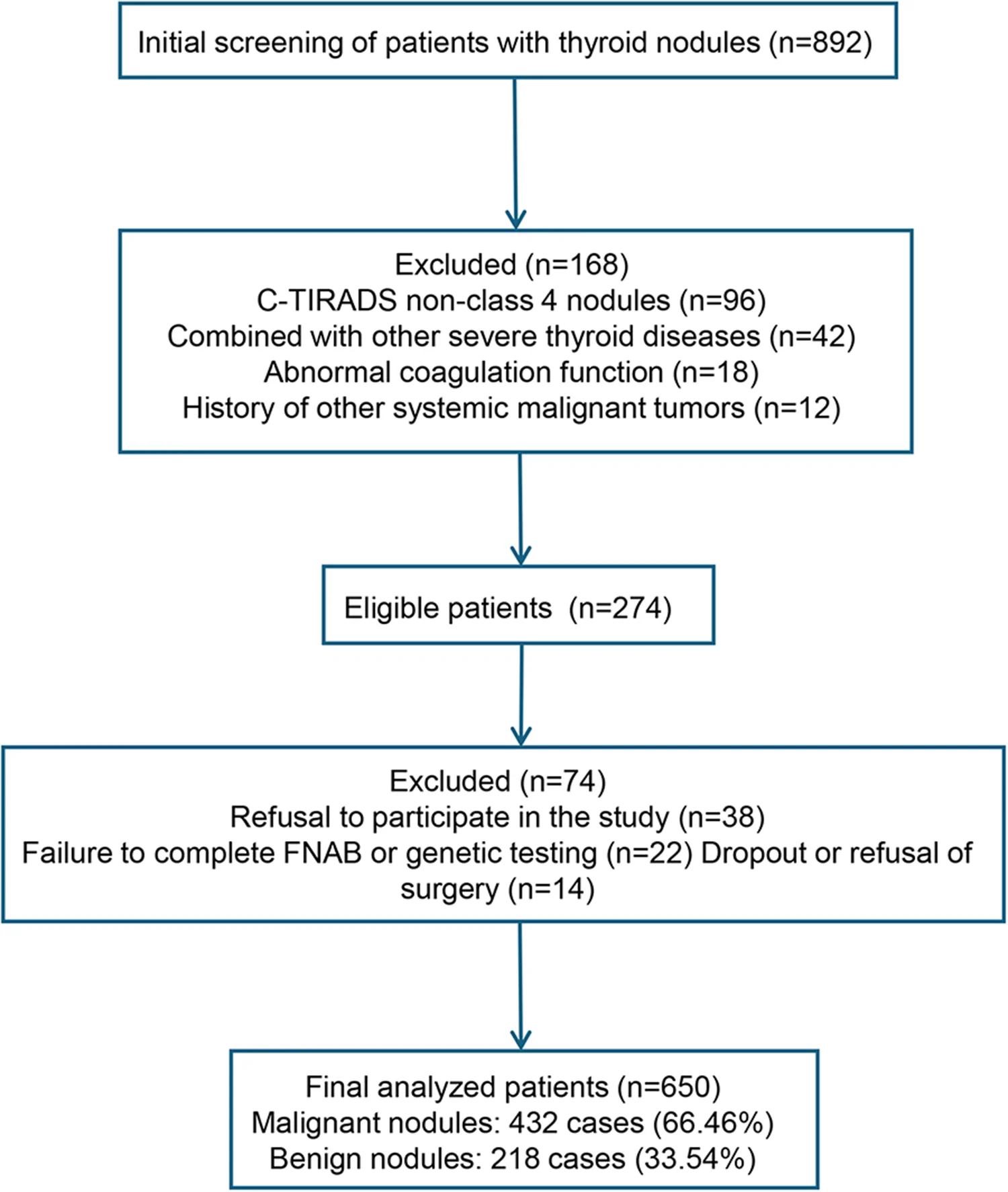
Patient enrollment flowchart.

### Distribution of gene mutations in different pathological types

As shown in Table 2, the distribution of gene mutations varied significantly among the different pathological types of thyroid nodules. In papillary thyroid carcinoma, BRAF^V600E^ was the most common mutation (342/386, 88.60%), followed by TERT (96/386, 24.87%) and RAS (28/386, 7.25%) mutations. Notably, 82 cases of papillary thyroid carcinoma (21.24%) harbored both BRAF^V600E^ and TERT mutations. In follicular thyroid carcinoma, RAS mutations were predominant (22/28, 78.57%), while BRAF^V600E^ mutations were absent. In medullary thyroid carcinoma, none of the four tested mutations were detected in most of the cases. In anaplastic carcinoma, TERT mutations were present in four of six cases (66.67%). Among benign nodules, BRAF^V600E^ mutations were found in 4 of 218 benign nodules (1.83%), RAS mutations in 8 nodules (3.67%), and TERT mutations and RET/PTC rearrangements were absent.

**Table 2:** Distribution of gene mutations across different pathological types.

Pathological type	*N*	BRAF^V600E^ (%)	TERT (%)	RAS (%)	RET/PTC (%)
Malignant nodules	432	342 (79.17)	106 (24.54)	52 (12.04)	28 (6.48)
Papillary thyroid carcinoma	386	342 (88.60)	96 (24.87)	28 (7.25)	26 (6.74)
Follicular thyroid carcinoma	28	0 (0)	6 (21.43)	22 (78.57)	0 (0)
Medullary thyroid carcinoma	12	0 (0)	0 (0)	2 (16.67)	2 (16.67)
Anaplastic carcinoma	6	0 (0)	4 (66.67)	0 (0)	0 (0)
Benign nodules	218	4 (1.83)	0 (0)	8 (3.67)	0 (0)
Nodular goiter	112	0 (0)	0 (0)	2 (1.79)	0 (0)
Thyroid adenoma	68	4 (5.88)	0 (0)	6 (8.82)	0 (0)
Hashimoto's thyroiditis	22	0 (0)	0 (0)	0 (0)	0 (0)
Subacute thyroiditis	16	0 (0)	0 (0)	0 (0)	0 (0)

### Comparison of the FNAB method and pathological diagnosis results

As shown in Table 3, FNAB alone detected 386 malignant thyroid nodules and 264 benign thyroid nodules, with a kappa value of 0.758.

**Table 3:** Comparison of the FNAB method and pathological diagnosis results.

Diagnostic method	Result	Pathological diagnosis		Total	Kappa
		Malignant	Benign		
FNAB	Malignant	338	48	386	0.758
	Benign	94	170	264	
Total		432	218	650	

### Comparison of single-gene testing methods

As shown in Table 4, the diagnostic performances of the individual gene tests varied considerably. BRAF^V600E^ testing alone demonstrated excellent specificity (98.17%) but moderate sensitivity (79.17%), missing 90 malignant nodules, including all 28 follicular thyroid carcinomas. TERT testing alone showed low sensitivity (24.54%) but 100% specificity, as no TERT mutations were detected in the benign nodules. RAS testing alone had a low sensitivity (12.04%) but relatively high specificity (96.33%), with eight false-positive cases among benign nodules because RAS mutations can also occur in benign follicular adenomas.

**Table 4:** Comparison of single-gene testing methods and pathological diagnosis results.

Diagnostic method	Result	Pathological diagnosis		Total	Sensitivity (%)	Specificity (%)
		Malignant	Benign			
BRAF^V600E^	Positive	342	4	346	79.17	98.17
	Negative	90	214	304		
TERT	Positive	106	0	106	24.54	100
	Negative	326	218	544		
RAS	Positive	52	8	60	12.04	96.33
	Negative	380	210	590		
RET/PTC	Positive	28	0	28	6.48	100
	Negative	404	218	622		
Multi-gene (any positive)	Positive	356	46	402	82.41	78.90
	Negative	76	172	248		

### Comparison of FNAB combined with single-gene testing methods

As shown in Table 5, FNAB combined with BRAF^V600E^ testing achieved a sensitivity of 87.96% (380/432), specificity of 77.06% (168/218), and an accuracy of 84.31%. Compared with FNAB alone, the addition of BRAF^V600E^ improved sensitivity (from 78.24% to 87.96%) but slightly reduced specificity (from 77.98% to 77.06%), consistent with the OR rule. FNAB combined with TERT testing achieved a sensitivity of 84.26% (364/432), specificity of 77.98% (170/218), and an accuracy of 82.15%. TERT testing helped identify 26 additional malignant cases that were missed by FNAB alone. FNAB combined with RAS testing achieved a sensitivity of 81.02% (350/432), specificity of 76.61% (167/218), and an accuracy of 79.54%. RAS testing helped detect 12 additional malignant cases, predominantly follicular thyroid carcinomas, but slightly reduced specificity because of RAS mutations in the benign nodules. FNAB combined with RET/PTC testing achieved a sensitivity of 80.09% (346/432), specificity of 77.98% (170/218), and an accuracy of 79.38%. However, the improvement was limited because RET/PTC rearrangements were present in only 28 malignant cases.

**Table 5:** Comparison of FNAB combined with single-gene testing methods.

Diagnostic method	TP	FP	FN	TN	Sensitivity (%)	Specificity (%)	Accuracy (%)	PPV (%)	NPV (%)
FNAB alone	338	48	94	170	78.24	77.98	78.15	87.56	64.39
FNAB + BRAF^V600E^	380	50	52	168	87.96	77.06	84.31	88.37	76.36
FNAB + TERT	364	48	68	170	84.26	77.98	82.15	88.35	71.43
FNAB + RAS	350	51	82	167	81.02	76.61	79.54	87.28	67.07
FNAB + RET/PTC	346	48	86	170	80.09	77.98	79.38	87.82	66.41
FNAB + multi-gene	393	52	39	166	91.00	76.15	86.00	88.31	80.98

TP, true positive; FP, false positive; FN, false negative; TN, true negative; PPV, positive predictive value; NPV, negative predictive value.

### Comparison of the multi-gene testing method and pathological diagnosis results

As shown in Table 6, multi-gene testing alone (positivity for any of BRAF^V600E^, TERT, RAS, or RET/PTC) detected 402 positive and 248 negative cases, with a sensitivity of 82.41%, specificity of 78.90%, and a kappa value of 0.772.

**Table 6:** Comparison of the multi-gene testing method and pathological diagnosis results.

Diagnostic method	Result	Pathological diagnosis		Total	Kappa
		Malignant	Benign		
Multi-gene testing	Positive	356	46	402	0.772
	Negative	76	172	248	
Total		432	218	650	

Positivity for multi-gene testing is defined as mutation positivity in any of BRAF^V600E^, TERT, RAS, or RET/PTC.

### Comparison of FNAB combined with the multi-gene testing method and pathological diagnosis results

As shown in Table 7, FNAB combined with multi-gene testing detected 445 positive and 205 negative cases. The sensitivity was 91.0% (393/432), specificity was 76.15% (166/218), and accuracy was 86.0% (559/650), with a kappa value of 0.681. Compared with FNAB alone, the combined test had higher sensitivity (91.0% vs. 78.24%) but slightly lower specificity (76.15% vs. 77.98%), consistent with the OR combination rule.

**Table 7:** Comparison of FNAB combined with the multi-gene testing method and pathological diagnosis results.

Diagnostic method	Result	Pathological diagnosis		Total	Kappa
		Malignant	Benign		
FNAB + multi-gene	Positive	393	52	445	0.681
	Negative	39	166	205	
Total		432	218	650	

### Summary comparison of all diagnostic methods

As shown in Table 8, a head-to-head comparison of all diagnostic approaches revealed distinct performance characteristics. Among FNAB combined with single-gene tests, FNAB + BRAF^V600E^ achieved the highest sensitivity (87.96%) and accuracy (84.31%) among the single-gene combinations, while FNAB + TERT showed the best specificity (77.98%). FNAB + RAS was the most effective method for detecting follicular thyroid carcinomas, but it had lower specificity because of RAS mutations in benign nodules. FNAB + RET/PTC provided the least improvement over FNAB alone because of the low prevalence of RET/PTC rearrangements.

**Table 8:** Summary comparison of all diagnostic methods for C-TIRADS 4 thyroid nodules (%).

Diagnostic method	Sensitivity (%)	Specificity (%)	Accuracy (%)	PPV (%)	NPV (%)
FNAB alone	78.24	77.98	78.15	87.56	64.39
BRAF^V600E^ alone	79.17	98.17	85.54	98.84	70.39
TERT alone	24.54	100	49.85	100	40.07
RAS alone	12.04	96.33	40.31	86.67	35.59
RET/PTC alone	6.48	100	37.85	100	35.05
Multi-gene alone	82.41	78.90	81.23	88.56	69.35
FNAB + BRAF^V600E^	87.96	77.06	84.31	88.37	76.36
FNAB + TERT	84.26	77.98	82.15	88.35	71.43
FNAB + RAS	81.02	76.61	79.54	87.28	67.07
FNAB + RET/PTC	80.09	77.98	79.38	87.82	66.41
FNAB + multi-gene	91.00	76.15	86.00	88.31	80.98

Importantly, FNAB combined with multi-gene testing achieved a sensitivity of 91.0%, specificity of 76.15%, and an accuracy of 86.0%. The AUC was 0.836, significantly higher than FNAB alone (0.781) and multi-gene testing alone (0.806) (*P* < 0.05). However, the specificity was not improved compared with FNAB alone, as expected under the OR rule.

### Performance of different diagnostic methods in FNAB-indeterminate nodules

Subgroup analysis was performed for patients with FNAB-indeterminate results (Bethesda III and IV). As shown in Table 9, among the 142 patients with indeterminate cytology (Bethesda III: 86 patients; Bethesda IV: 56 patients), postoperative pathology confirmed malignancy in 68 cases (47.89%). In this challenging subgroup, FNAB alone (using Bethesda V/VI as positive) had a sensitivity of 0% because all 142 nodules were Bethesda III/IV and therefore negative by this definition.

**Table 9:** Diagnostic performance in FNAB-indeterminate nodules (Bethesda III/IV, *n* = 142).

Diagnostic method	TP	FP	FN	TN	Sensitivity (%)	Specificity (%)	Accuracy (%)
FNAB (Bethesda V/VI+)	0	0	68	74	0	100	52.11
FNAB + BRAF^V600E^	50	3	18	71	73.53	95.95	85.21
FNAB + TERT	35	0	33	74	51.47	100	76.76
FNAB + RAS	48	7	20	67	70.59	90.54	80.99
FNAB + RET/PTC	6	0	62	74	8.82	100	56.34
FNAB + multi-gene	60	8	8	66	88.24	89.19	88.73

Among the single-gene tests combined with FNAB, FNAB + BRAF^V600E^ achieved a sensitivity of 73.53% and specificity of 95.95%. FNAB + RAS was particularly valuable for detecting follicular-patterned malignancies, increasing the sensitivity to 70.59%, but with lower specificity (90.54%). FNAB + TERT helped identify high-risk cases (sensitivity, 51.47%; specificity; 100%). FNAB + RET/PTC provided limited additional diagnostic value in this subgroup, with a sensitivity of 8.82% and a specificity of 100%.

Notably, FNAB combined with multi-gene testing achieved improved sensitivity in indeterminate nodules, with a sensitivity of 88.24%, specificity of 89.19%, and an accuracy of 88.73%. Although the specificity was lower than that of FNAB alone (100%), the combined approach significantly improved the sensitivity and overall accuracy (*P* < 0.01).

### Sensitivity analysis reclassifying Bethesda IV as FNAB-positive

Among the 56 Bethesda IV nodules, postoperative pathology confirmed 23 malignant and 33 benign nodules. When Bethesda IV was reclassified as FNAB-positive, FNAB alone achieved a sensitivity of 83.6% (361/432), specificity of 62.8% (137/218), accuracy of 76.6% (498/650), positive predictive value of 81.7% (361/442), negative predictive value of 65.9% (137/208), and AUC of 0.732 (95% CI: 0.691–0.773). The results are summarized in Table 10. As expected, this reclassification increased the sensitivity of FNAB alone but substantially reduced its specificity, and the overall diagnostic discrimination was lower than that of FNAB combined with multi-gene testing.

**Table 10:** Sensitivity analysis for FNAB alone with Bethesda IV reclassified as FNAB-positive.

Method	Sensitivity (%)	Specificity (%)	Accuracy (%)	PPV (%)	NPV (%)	AUC
FNAB alone (primary analysis: Bethesda IV negative)	78.24	77.98	78.15	87.56	64.39	0.781
FNAB alone (sensitivity analysis: Bethesda IV positive)	83.6	62.8	76.6	81.7	65.9	0.732

In the sensitivity analysis, 56 Bethesda IV nodules were reclassified as FNAB-positive. Among these 56 nodules, 23 were malignant and 33 were benign according to postoperative pathology.

### ROC curve comparison of the three methods for diagnosing C-TIRADS 4 thyroid nodules

As shown in Figure 2, the ROC curves of the different diagnostic methods were plotted on the same graph for comparison. The AUC of FNAB alone was 0.781 (95% CI: 0.742–0.820). Among the single-gene tests, BRAF^V600E^ alone achieved an AUC of 0.886 (95% CI: 0.860–0.912), TERT alone had an AUC of 0.622 (95% CI: 0.580–0.665), RAS alone had an AUC of 0.541 (95% CI: 0.496–0.587), and RET/PTC alone had an AUC of 0.532 (95% CI: 0.486–0.578). Multi-gene testing alone achieved an AUC of 0.806 (95% CI: 0.768–0.844).

**Figure 2:**
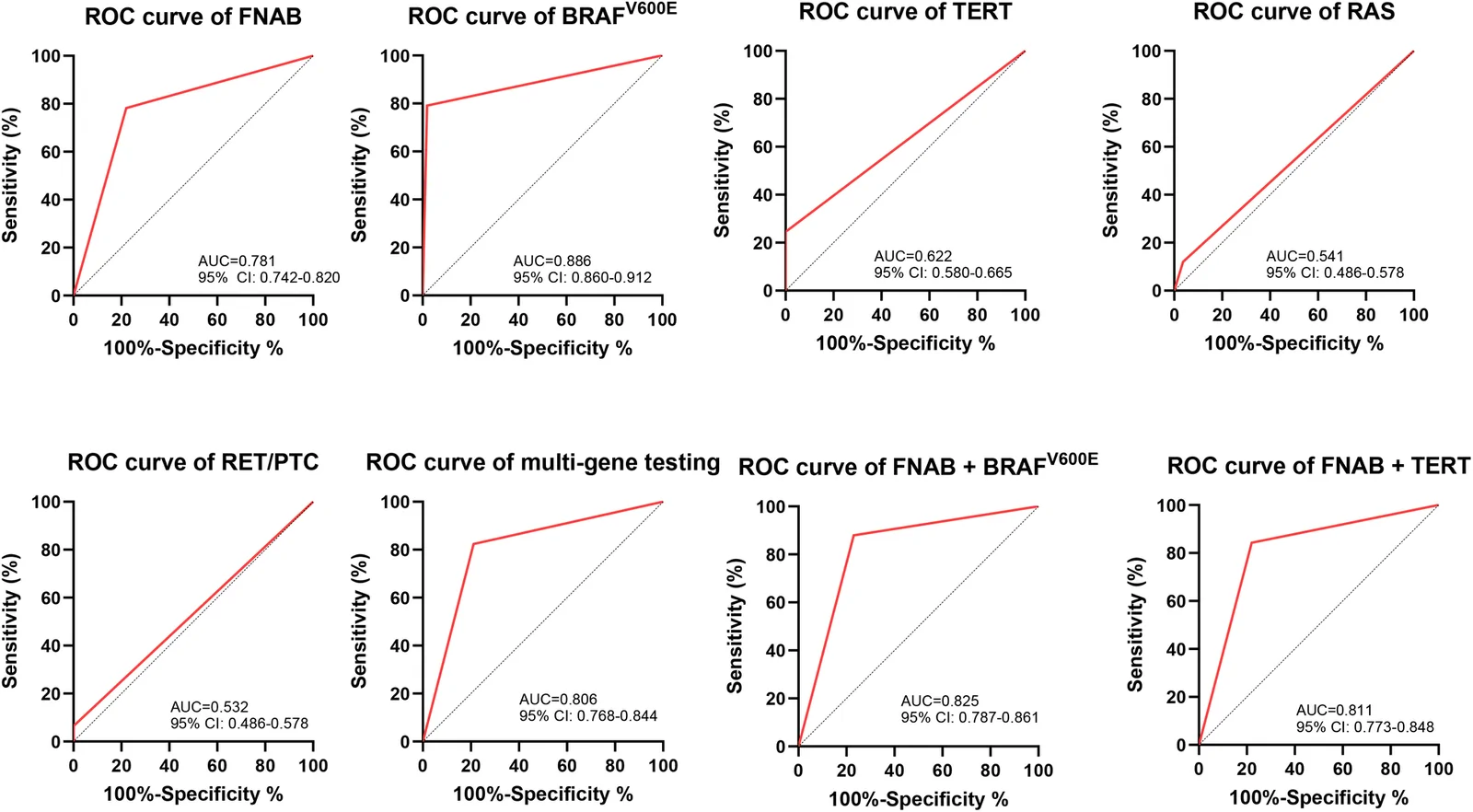
ROC curves of different diagnostic methods for C-TIRADS 4 thyroid nodules.

Among the combination approaches, FNAB + BRAF^V600E^ achieved an AUC of 0.825, FNAB + TERT achieved 0.811, FNAB + RAS achieved 0.788, and FNAB + RET/PTC achieved 0.790. FNAB + multi-gene testing achieved an AUC of 0.836. Because all diagnostic tests were binary, the ROC curves represented single operating points, and AUCs were interpreted as summary measures of discrimination. DeLong test comparisons showed that the AUC of FNAB combined with multi-gene testing was significantly higher than that of FNAB alone (*P* < 0.001), multi-gene testing alone (*P* = 0.003), FNAB + TERT (*P* = 0.008), FNAB + RAS (*P* = 0.002), and FNAB + RET/PTC (*P* = 0.004), but not significantly different from that of FNAB + BRAFV600E (*P* = 0.214).

### Typical case of pathological images

Figure 3 shows a typical case of a patient with a C-TIRADS 4B nodule diagnosed with papillary thyroid carcinoma by FNAB combined with multi-gene testing. Preoperative FNAB cytology suggested Bethesda grade V (suspicious for malignancy), with concurrent positive BRAF^V600E^ gene mutation testing, and postoperative pathology confirmed papillary thyroid carcinoma.

**Figure 3:**
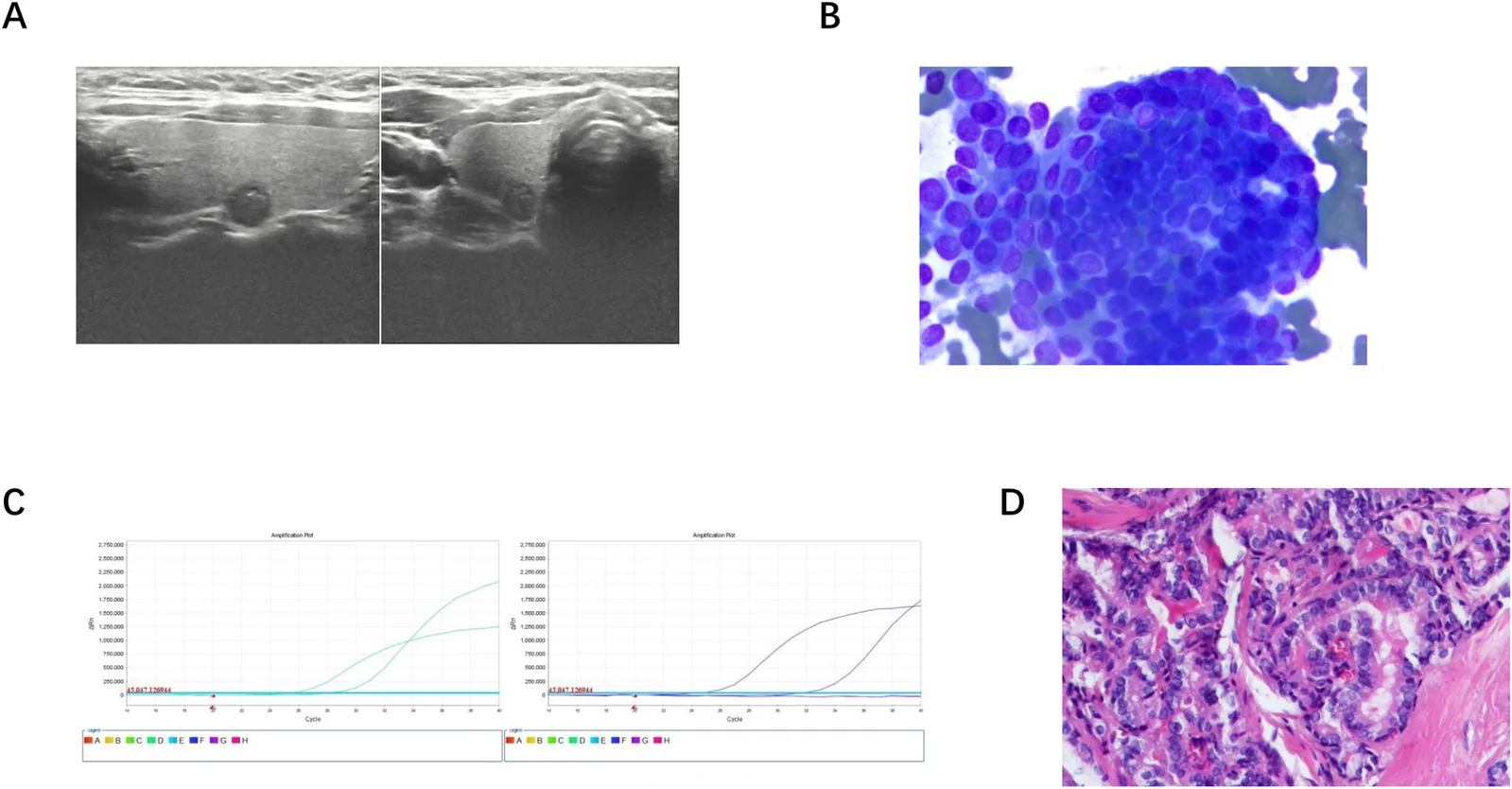
Pathological images of a typical case. **(A)** Ultrasound image showed a solid hypoechoic nodule with indistinct borders, and microcalcification is seen in the middle and posterior-medial parts of the right lobe of the thyroid; **(B)** FNAB cell smear showed enlarged cell nuclei, prominent nuclear grooves, and intranuclear pseudouni-cellular inclusions (Bethesda grade V, ×400); **(C)** The BRAF^V600E^ gene amplification curve shows a positive result; **(D)** Postoperative pathological HE staining showed a typical papillary structure (×200).

## Discussion

C-TIRADS provides a standardized framework for thyroid nodule risk stratification; however, category 4 nodules remain diagnostically difficult because of the overlapping ultrasound features between benign and malignant lesions (13, 14). In the present study, FNAB alone demonstrated moderate diagnostic performance, with a sensitivity of 78.24% and a specificity of 77.98%. This is consistent with previous reports, indicating that cytology alone results in a substantial proportion of indeterminate diagnoses (15). This limitation provides a rationale for integrating molecular testing into the diagnostic pathway.

Among the individual markers, BRAF^V600E^ shows excellent specificity but lower sensitivity because it is largely restricted to papillary thyroid carcinoma and is absent in follicular and medullary carcinomas (16). TERT and RET/PTC also demonstrated high specificity but low sensitivity, whereas RAS exhibited moderate sensitivity but lower specificity due to its presence in some benign follicular lesions. These findings reflect both the intrinsic biology of each marker and the histological composition of the study cohort. Importantly, the diagnostic performance of each marker should not be interpreted as fixed because it is influenced by the proportion of specific tumor subtypes in the study population.

Multi-gene testing alone had higher sensitivity than FNAB alone but similar specificity, mainly because it detected additional BRAF-negative malignancies, such as follicular carcinoma and selected high-risk papillary carcinomas. When FNAB was combined with multi-gene testing using the OR rule, the sensitivity improved to 91.0%, but specificity was 76.15%, slightly lower than that of FNAB alone (77.98%). This is an expected consequence of the OR combination rule and indicates that the main benefit of the combined strategy is improved sensitivity, rather than improved specificity. The AUC of the combined approach was 0.836, significantly higher than that of FNAB alone and multi-gene testing alone, but not significantly different from FNAB + BRAF^V600E^ (AUC = 0.825). Therefore, the choice between single-gene and multi-gene testing should consider the specific clinical question and case mix.

In the subgroup with FNAB-indeterminate cytology (Bethesda III/IV), FNAB combined with multi-gene testing achieved a sensitivity of 88.24% and a specificity of 89.19%. Although specificity was lower than that of FNAB alone in this subgroup, the marked improvement in sensitivity suggests that multi-gene testing may help reclassify indeterminate nodules, particularly those with follicular-patterned morphology or non-BRAF mutations. These observations should be interpreted as hypothesis-generating and require validation in future prospective studies.

From a clinical applicability perspective, the present findings should not be interpreted as supporting routine multi-gene testing for all C-TIRADS 4 nodules. C-TIRADS 4 includes heterogeneous subgroups (4A/4B/4C), and the additional cost of universal testing is unlikely to be justified without a selective application. Molecular testing is likely to be most useful in patients with indeterminate cytology, discordant imaging–cytology findings, or suspicion of non-PTC subtypes. However, the present retrospective design cannot establish clinical benefits or cost-effectiveness, and these issues require dedicated studies.

This study has several limitations. First, this was a single-center retrospective study, which limited its generalizability. Second, the multi-gene panel did not include additional relevant alterations, such as PTEN, PAX8/PPAR*γ*, or NTRK fusions. Third, the analysis combined the C-TIRADS 4A, 4B, and 4C subgroups, which may have introduced heterogeneity. Fourth, rare tumor subtypes were limited in number, precluding subtype-specific analysis. Finally, the ROC curves were based on binary test results, and therefore, the AUC values should be interpreted as summary measures rather than threshold-independent indices.

## Conclusion

In this retrospective cohort study, FNAB combined with multi-gene testing (BRAF^V600E^, TERT, RAS, and RET/PTC) improved diagnostic sensitivity and overall discrimination for C-TIRADS 4 thyroid nodules compared to FNAB alone or multi-gene testing alone. However, the specificity was not improved because of the OR combination rule. The integrated strategy may be most valuable in selected patients, particularly those with FNAB-indeterminate cytology or BRAF-negative malignancies, but it should not be considered a routine requirement for all C-TIRADS 4 nodules. Prospective multicenter studies are needed to validate these findings and define the optimal selection criteria for molecular testing.

## Data Availability

The original contributions presented in this study are included in the article/Supplementary Material. Further inquiries can be directed to the corresponding author.
